# Distinct clinical symptom patterns in patients hospitalised with COVID-19 in an analysis of 59,011 patients in the ISARIC-4C study

**DOI:** 10.1038/s41598-022-08032-3

**Published:** 2022-04-27

**Authors:** Jonathan E. Millar, Lucile Neyton, Sohan Seth, Jake Dunning, Laura Merson, Srinivas Murthy, Clark D. Russell, Sean Keating, Maaike Swets, Carole H. Sudre, Timothy D. Spector, Sebastien Ourselin, Claire J. Steves, Jonathan Wolf, Annemarie B. Docherty, Ewen M. Harrison, Peter J. M. Openshaw, Malcolm G. Semple, J. Kenneth Baillie, ISARIC-4C, J. Kenneth Baillie, Malcolm G. Semple, Peter J. M. Openshaw, Gail Carson, Beatrice Alex, Benjamin Bach, Wendy S. Barclay, Debby Bogaert, Meera Chand, Graham S. Cooke, Annemarie B. Docherty, Jake Dunning, Anna da Silva Filipe, Tom Fletcher, Christopher A. Green, Ewen M. Harrison, Julian A. Hiscox, Antonia YW Ho, Peter W. Horby, Samreen Ijaz, Saye Khoo, Paul Klenerman, Andrew Law, Wei Shen Lim, Alexander J. Mentzer, Laura Merson, Alison M. Meynert, Mahdad Noursadeghi, Shona C. Moore, Massimo Palmarini, William A. Paxton, Georgios Pollakis, Nicholas Price, Andrew Rambaut, David L. Robertson, Clark D. Russell, Vanessa Sancho-Shimizu, Janet T. Scott, Louise Sigfrid, Tom Solomon, Shiranee Sriskandan, David Stuart, Charlotte Summers, Richard S. Tedder, Emma C. Thomson, Ryan S. Thwaites, Lance C. W. Turtle, Maria Zambon, Hayley Hardwick, Chloe Donohue, Jane Ewins, Wilna Oosthuyzen, Fiona Griffiths, Lisa Norman, Riinu Pius, Tom M. Drake, Cameron J. Fairfield, Stephen Knight, Kenneth A. Mclean, Derek Murphy, Catherine A. Shaw, Jo Dalton, Michelle Girvan, Egle Saviciute, Stephanie Roberts, Janet Harrison, Laura Marsh, Marie Connor, Gary Leeming, Ross Hendry, William Greenhalf, Victoria Shaw, Sarah McDonald, Kayode Adeniji, Daniel Agranoff, Ken Agwuh, Dhiraj Ail, Ana Alegria, Brian Angus, Abdul Ashish, Dougal Atkinson, Shahedal Bari, Gavin Barlow, Stella Barnass, Nicholas Barrett, Christopher Bassford, David Baxter, Michael Beadsworth, Jolanta Bernatoniene, John Berridge, Nicola Best, Pieter Bothma, David Brealey, Robin Brittain-Long, Naomi Bulteel, Tom Burden, Andrew Burtenshaw, Vikki Caruth, David Chadwick, Duncan Chambler, Nigel Chee, Jenny Child, Srikanth Chukkambotla, Tom Clark, Paul Collini, Graham Cooke, Catherine Cosgrove, Jason Cupitt, Maria-Teresa Cutino-Moguel, Paul Dark, Chris Dawson, Samir Dervisevic, Phil Donnison, Sam Douthwaite, Ingrid DuRand, Ahilanadan Dushianthan, Tristan Dyer, Cariad Evans, Chi Eziefula, Chrisopher Fegan, Adam Finn, Duncan Fullerton, Sanjeev Garg, Atul Garg, Jo Godden, Arthur Goldsmith, Clive Graham, Elaine Hardy, Stuart Hartshorn, Daniel Harvey, Peter Havalda, Daniel B. Hawcutt, Maria Hobrok, Luke Hodgson, Anita Holme, Anil Hormis, Michael Jacobs, Susan Jain, Paul Jennings, Agilan Kaliappan, Vidya Kasipandian, Stephen Kegg, Michael Kelsey, Jason Kendall, Caroline Kerrison, Ian Kerslake, Oliver Koch, Gouri Koduri, George Koshy, Shondipon Laha, Susan Larkin, Tamas Leiner, Patrick Lillie, James Limb, Vanessa Linnett, Jeff Little, Michael MacMahon, Emily MacNaughton, Ravish Mankregod, Huw Masson, Elijah Matovu, Katherine McCullough, Ruth McEwen, Manjula Meda, Gary Mills, Jane Minton, Mariyam Mirfenderesky, Kavya Mohandas, Quen Mok, James Moon, Elinoor Moore, Patrick Morgan, Craig Morris, Katherine Mortimore, Samuel Moses, Mbiye Mpenge, Rohinton Mulla, Michael Murphy, Megan Nagel, Thapas Nagarajan, Mark Nelson, Igor Otahal, Mark Pais, Selva Panchatsharam, Hassan Paraiso, Brij Patel, Justin Pepperell, Mark Peters, Mandeep Phull, Stefania Pintus, Jagtur Singh Pooni, Frank Post, David Price, Rachel Prout, Nikolas Rae, Henrik Reschreiter, Tim Reynolds, Neil Richardson, Mark Roberts, Devender Roberts, Alistair Rose, Guy Rousseau, Brendan Ryan, Taranprit Saluja, Aarti Shah, Prad Shanmuga, Anil Sharma, Anna Shawcross, Jeremy Sizer, Richard Smith, Catherine Snelson, Nick Spittle, Nikki Staines, Tom Stambach, Richard Stewart, Pradeep Subudhi, Tamas Szakmany, Kate Tatham, Jo Thomas, Chris Thompson, Robert Thompson, Ascanio Tridente, Darell Tupper-Carey, Mary Twagira, Andrew Ustianowski, Nick Vallotton, Lisa Vincent-Smith, Shico Visuvanathan, Alan Vuylsteke, Sam Waddy, Rachel Wake, Andrew Walden, Ingeborg Welters, Tony Whitehouse, Paul Whittaker, Ashley Whittington, Meme Wijesinghe, Martin Williams, Lawrence Wilson, Sarah Wilson, Stephen Winchester, Martin Wiselka, Adam Wolverson, Daniel G. Wooton, Andrew Workman, Bryan Yates, Peter Young

**Affiliations:** 1 Division of Functional Genetics and Development, Roslin Institute, University of Edinburgh, Easter Bush Campus, Midlothian, Edinburgh, EH25 9RG UK; 2 Institute for Adaptive and Neural Computation, School of Informatics, University of Edinburgh, Edinburgh, UK; 3 National Infection Service, Public Health England, London, UK; 4 National Heart and Lung Institute, Imperial College London, London, UK; 5 Centre for Tropical Medicine and Global Health, Nuffield Department of Medicine, ISARIC Global Support Centre, University of Oxford, Oxford, UK; 6 Infectious Diseases Data Observatory, Centre for Tropical Medicine and Global Health, University of Oxford, Oxford, UK; 7 BC Children’s Hospital, University of British Columbia, Vancouver, Canada; 8 Centre for Inflammation Research, The Queen’s Medical Research Institute, University of Edinburgh, Edinburgh, UK; 9 Intensive Care Unit, Royal Infirmary of Edinburgh, Edinburgh, UK; 10 Department of Infectious Diseases, Leiden University Medical Center, Leiden, The Netherlands; 11 School of Biomedical and Imaging Sciences, King’s College London, London, UK; 12 Department of Twin Research and Genetic Epidemiology, King’s College London, London, UK; 13 ZOE Global Ltd, London, UK; 14 Centre for Medical Informatics, Usher Institute, University of Edinburgh, Edinburgh, UK; 15 NIHR Health Protection Research Unit in Emerging and Zoonotic Infections, Institute of Infection, Veterinary and Ecological Sciences, Faculty of Health and Life Sciences, University of Liverpool, Liverpool, UK; 16 School of Informatics, University of Edinburgh, Edinburgh, UK; 17 Section of Molecular Virology, Imperial College London, London, UK; 18 Antimicrobial Resistance and Hospital Acquired Infection Department, Public Health England, London, UK; 19 Department of Infectious Disease, Imperial College London, London, UK; 20 Centre for Virus Research, Sir Michael Stoker Building, University of Glasgow, Glasgow, UK; 21 Liverpool School of Tropical Medicine, Liverpool, UK; 22 Institute of Microbiology and Infection, University of Birmingham, Birmingham, UK; 23 Institute of Infection, Veterinary and Ecological Sciences, University of Liverpool, Liverpool, UK; 24 MRC-University of Glasgow Centre for Virus Research, University of Glasgow, Glasgow, UK; 25 Centre for Tropical Medicine and Global Health, Nuffield Department of Medicine, University of Oxford, Oxford, UK; 26 Virology Reference Department, National Infection Service, Public Health England, London, UK; 27 Department of Pharmacology, University of Liverpool, Liverpool, UK; 28 Nuffield Department of Medicine, Peter Medawar Building for Pathogen Research, University of Oxford, Oxford, UK; 29 Nottingham University Hospitals NHS Trust, Nottingham, UK; 30 Nuffield Department of Medicine, John Radcliffe Hospital, Oxford, UK; 31 MRC Human Genetics Unit, MRC, Institute of Genetics and Molecular Medicine, University of Edinburgh, Edinburgh, UK; 32 Division of Infection and Immunity, University College London, London, UK; 33 Institute of Infection and Global Health, University of Liverpool, Liverpool, UK; 34 Centre for Clinical Infection and Diagnostics Research, Department of Infectious Diseases, School of Immunology and Microbial Sciences, King’s College London, London, UK; 35 Institute of Evolutionary Biology, University of Edinburgh, Edinburgh, UK; 36 Department of Pediatrics and Virology, St Mary’s Medical School Bldg, Imperial College London, London, UK; 37 Division of Structural Biology, The Wellcome Centre for Human Genetics, University of Oxford, Oxford, UK; 38 Department of Medicine, University of Cambridge, Cambridge, Cambridgeshire UK; 39 Blood Borne Virus Unit, Virus Reference Department, National Infection Service, Public Health England, London, UK; 40 Public Health England, London, UK; 41 ISARIC 4C Management Team, C/O Roslin Institute, University of Edinburgh, Edinburgh, UK

**Keywords:** Signs and symptoms, Infectious diseases

## Abstract

COVID-19 is clinically characterised by fever, cough, and dyspnoea. Symptoms affecting other organ systems have been reported. However, it is the clinical associations of different patterns of symptoms which influence diagnostic and therapeutic decision-making. In this study, we applied clustering techniques to a large prospective cohort of hospitalised patients with COVID-19 to identify clinically meaningful sub-phenotypes. We obtained structured clinical data on 59,011 patients in the UK (the ISARIC Coronavirus Clinical Characterisation Consortium, 4C) and used a principled, unsupervised clustering approach to partition the first 25,477 cases according to symptoms reported at recruitment. We validated our findings in a second group of 33,534 cases recruited to ISARIC-4C, and in 4,445 cases recruited to a separate study of community cases. Unsupervised clustering identified distinct sub-phenotypes. First, a core symptom set of fever, cough, and dyspnoea, which co-occurred with additional symptoms in three further patterns: fatigue and confusion, diarrhoea and vomiting, or productive cough. Presentations with a single reported symptom of dyspnoea or confusion were also identified, alongside a sub-phenotype of patients reporting few or no symptoms. Patients presenting with gastrointestinal symptoms were more commonly female, had a longer duration of symptoms before presentation, and had lower 30-day mortality. Patients presenting with confusion, with or without core symptoms, were older and had a higher unadjusted mortality. Symptom sub-phenotypes were highly consistent in replication analysis within the ISARIC-4C study. Similar patterns were externally verified in patients from a study of self-reported symptoms of mild disease. The large scale of the ISARIC-4C study enabled robust, granular discovery and replication. Clinical interpretation is necessary to determine which of these observations have practical utility. We propose that four sub-phenotypes are usefully distinct from the core symptom group: gastro-intestinal disease, productive cough, confusion, and pauci-symptomatic presentations. Importantly, each is associated with an in-hospital mortality which differs from that of patients with core symptoms.

## Introduction

Coronavirus-19 disease (COVID-19) is characterised by a triad of symptoms: cough, fever and dyspnoea. However, it is clear that COVID-19 is not a homogeneous clinical entity. Important biological differences exist between sub-phenotypes, as is seen in other forms of critical illness including sepsis^1^, pancreatitis^2^, and dengue^3^. Remarkably, this is already evident for COVID-19: highly significant sub-group effects have been seen in trials demonstrating the efficacy of dexamethasone^4,5^ and tocilizumab^6^, and progress has been made to uncover their underlying biology^7^. Similarly, genome-wide association studies have identified a genetic basis for differences in the host response to SARS-CoV-2 infection^8^.

The recognition of clinical similarities between patients is a fundamental unit of medical progress. Grouping patients enables us to select appropriate diagnostic tests, predict response to therapy, and to prognosticate. Symptoms of disease are the most basic characteristics available to clinicians for stratifying patients with any illness. Simple machine learning methods can reveal patterns in clinical symptoms^9^ with diagnostic and therapeutic relevance^10^.

The International Severe Acute Respiratory Infection Consortium Clinical Characterisation Study (ISARIC-4C) is an ongoing study of patients admitted to more than 260 acute hospitals in the United Kingdom. We employed unsupervised machine learning techniques in a large, prospective cohort of patients with SARS-CoV-2, admitted during the ‘first wave’, to better characterise the symptoms of COVID-19 and to identify sub-phenotypes based on symptoms.

## Results

For the initial cohort, we studied the first 33,468 patients enrolled to the ISARIC-4C study, of which 7,991 had fully missing symptom data and were excluded from our analysis (Figure S1). The baseline characteristics of included patients (n = 25,477) are detailed in Table S1. Overall, the median age was 73 (59–83) years and the majority of patients were male (n = 15,046 (59%)). On average, individuals presented to hospital 4 (1–8) days after the onset of symptoms.

### Symptom prevalence and the relationship between symptoms

**Figure 1:**
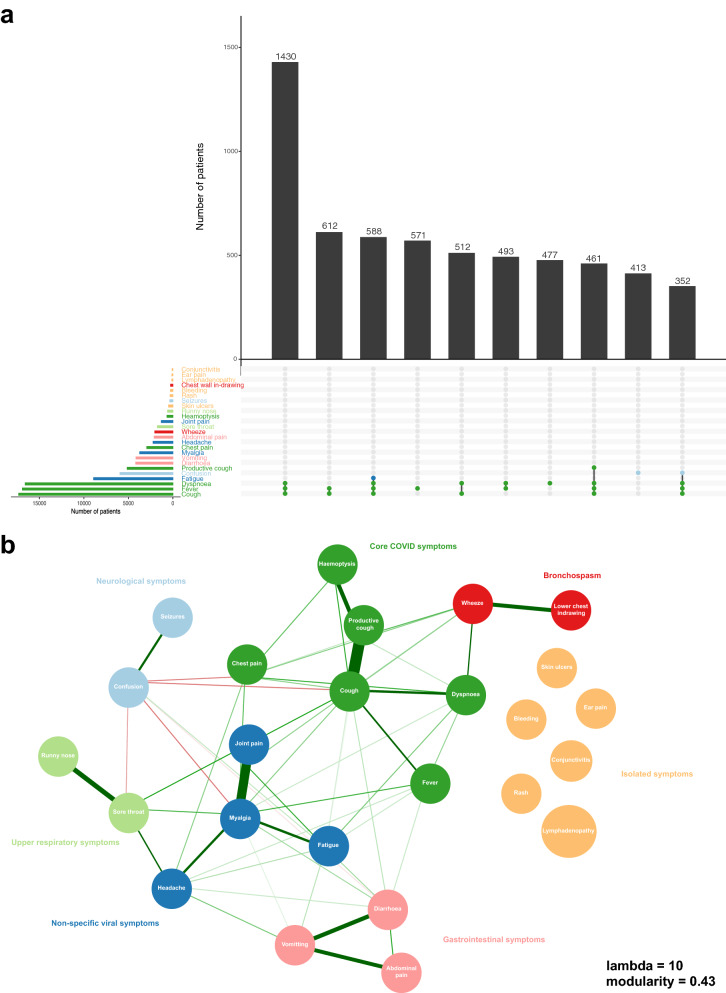
Symptom prevelence and relationships. (**a**) Symptom prevalence. Upset plot, intersections describe the ‘top 10’ symptom combinations within the cohort. The upper graph charts the total number of patients exhibiting these symptom sets. The lower graph charts the total number of patients with each symptom in the cohort. (**b**) Symptom network graph, derived using eLasso. Lines between symptom nodes illustrate conditional dependencies. A thicker line width and darker hue represents a stronger positive conditional dependence. Red lines represent a negative conditional dependence.

Cough was the most prevalent symptom (68.0%, 95% CI 67.5–68.6%), followed by fever (66.4%, 95% CI 65.8–67%), and dyspnoea (65.2%, 95% CI 64.6–65.8%) (Fig. 1a and Table S2). Furthermore, these were the only symptoms to be reported by more than half of participants. The prevalence of individual symptoms varied with age (Figure S2). Fever was less marked at the extremes of age, an observation which was also evident for dyspnoea, and, with the exception of those aged > 90 years, for cough. Similarly, rash and runny nose were limited mostly to those aged < 20 years, especially to those aged under 10 years. In sum, there were 4,335 unique symptom combinations in the cohort, the most frequent being fever, cough, and dyspnoea (n = 1,430 (5.6%)) (Fig. 1a).

To explore the relationships between symptoms, we fit an Ising model, employing L1-regularised logistic regression. The majority of symptoms exhibited some degree of conditional dependence with at least one other, however, there were several that occurred independently: skin ulcers, rash, bleeding, lymphadenopathy, ear pain, and conjunctivitis (Fig. 1b). All of which had a low prevalence (< 5%). Uniquely, confusion was negatively associated with cough, myalgia, sore throat, and diarrhoea. Groupings of symptoms with interconnected, positive conditional dependencies were appreciable from inspection of the network graph. To formalise communities, we used a short random walk algorithm. Excluding the 6 orphan symptoms, 6 distinct communities were identified. These include: core COVID-19 (fever, cough, and dyspnoea), upper respiratory, bronchospasm, gastrointestinal (GI), neurological, and non-specific viral symptom sets (Fig. 1b).

#### Symptom sub-phenotype derivation

To identify symptom sub-phenotypes within the study cohort, we performed unsupervised partitional clustering. An a priori assessment suggested that 7 clusters was the optimal solution by majority assessment. This combined the inflection points in the decline in total sum of squares and the rise in gap statistic, with the nearest peak in average silhouette width (Figure S3).

**Figure 2:**
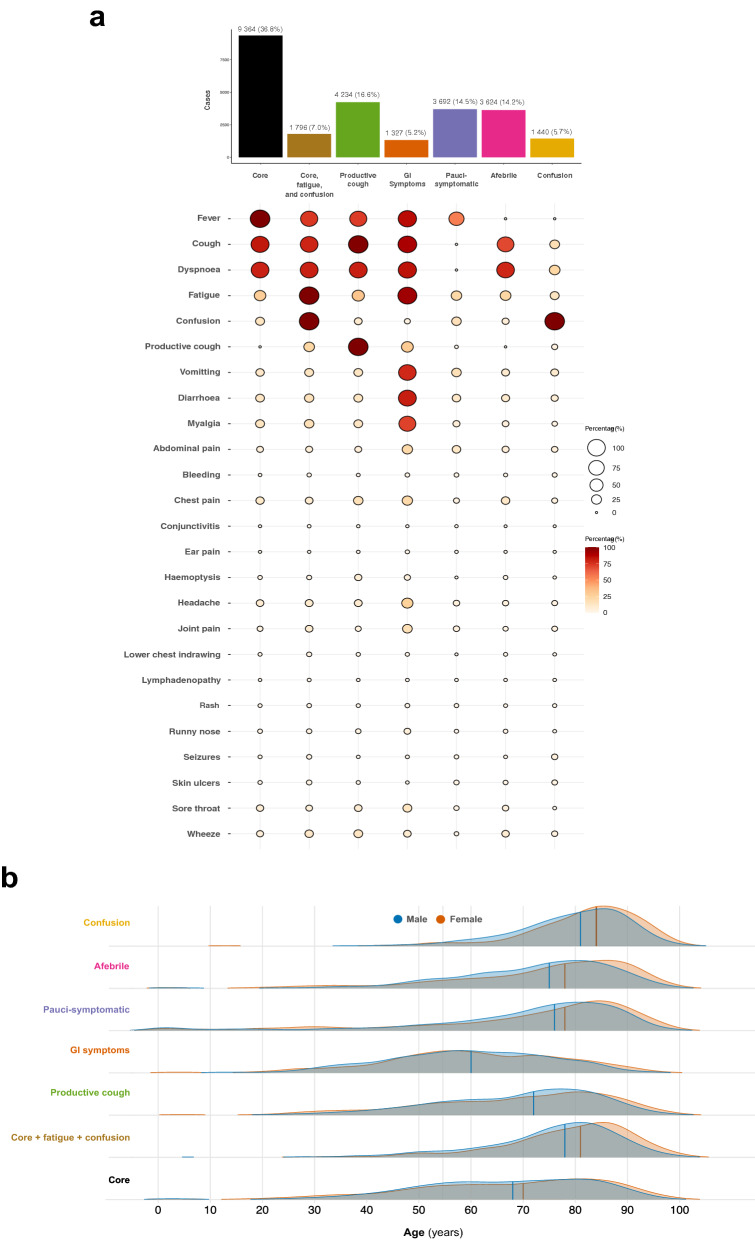
Symptom clusters. (**a**) Cluster identities, proportions, and patterns. Data are presented as count (percentage). (**b**) Distribution of age by symptom cluster. Density plots, solid lines represent the median age.

The patterns of symptoms within the seven clusters are shown in Fig. 2a. Based on the central (medoid) case, we characterised the sub-phenotypes as: core COVID-19 symptoms (fever, cough, and dyspnoea); core symptoms plus fatigue and confusion; productive cough; gastrointestinal (GI) symptoms; pauci-symptomatic (no single symptom being present in > 50% of the cluster membership); afebrile; and confusion. The core symptoms sub-phenotype accounted for the largest number of patients (n = 9,364 (36.8%)) and the GI symptoms sub-phenotype the fewest (n = 1,327 (5.2%)). Measures of internal validity and stability are presented in Figure S4a.

#### Sub-phenotype sensitivity analyses

To examine the implications of our handling of missing data, we performed a sensitivity analysis by clustering only cases with fully complete symptom data (n = 12,712 (49.9%)). This analysis retained the cluster structure, except for the afebrile sub-phenotype, in which the medoid case exhibited dyspnoea alone (Figure S4b). The simple agreement between iterations was 89.2%, with a Cohen's kappa of 0.86 (p < 0.001). Patients with fully missing data (n = 7,991) were of a similar age (74 (59–84) years) and sex (57.6% male). We conducted three additional analyses to explore the contribution of; age, heterogeneity of symptom recording, and the time from symptom onset to study enrolment. For age, we replicated our clustering pipeline in the subset of the primary cohort aged ≥ 70 years (Table S3. In this instance, in the absence of confusion as a discriminatory symptom, we remained able to retrieve the core, productive cough, GI symptoms, pauci-symptomatic, and afebrile sub-phenotypes. For heterogeneity of symptom recording, we grouped the clustered primary cohort by recruiting site (sites recruiting ≥ 50 patients, 122 sites, n = 24,279), and analysed the proportion of patients reporting each of the 9 most discriminatory symptoms by site (Figure S5. All, except for myalgia, had a co-efficient of variation < 0.35. Finally, for time from symptom onset to study enrolment, we divided the clustered primary cohort into its deciles. We then visualised the proportion of cases assigned to each sub-phenotype per decile (Figure S6). All sub-phenotypes were present in each decile.

#### Sub-phenotype replication

We replicated our clustering in two independent cohorts. First, we repeated our clustering approach on a cohort of patients enrolled to the ISARIC CCP-UK after those in the primary analysis and up until 7^th^ July, 2020 (n = 35,446). Of these, 1,912 (5.4%) had fully missing symptom data and were excluded from analysis. Clustering returned identical sub-phenotypes, with the exception of the afebrile cluster, in which cough was no longer implicated (Figure S7). This cluster was also reduced in relative size (14.2% to 6.7%). Second, our clustering approach was replicated in an outpatient cohort (COVID Symptom Study, n = 4,445). This study records several overlapping or closely associated symptoms. Despite differences in study design and population, similar sub-phenotypes are discernible, including GI (cluster 1), pauci-symptomatic (cluster 2), and confusion (cluster 5) (Figure S8).

#### Association of sub-phenotypes with patient characteristics

**Figure 3:**
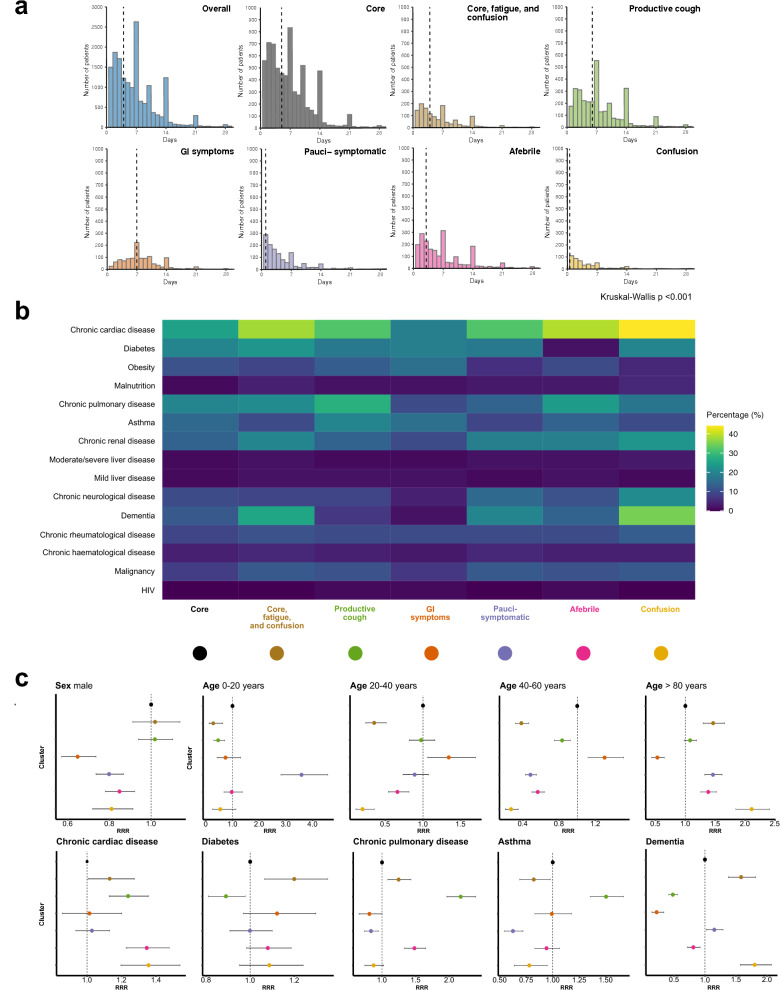
Patient characteristics and symptom clusters. (**a**) Time from symptom onset to hospital admission. Data are presented as counts in singleday bins. Vertical dashed lines represent the median time (days). (**b**) Co-morbidities by symptom cluster. Percentage of individuals with co-morbidity at time of admission. (**c**) Association between patient characteristics and symptom cluster membership. Multinomial regression, presented as relative risk ratio (95% confidence interval). Core symptoms chosen as reference cluster. The age group 60-80 years serves as the reference group for age.

Compared to the primary cohort average (73 years (59–83)), those in the GI symptoms sub-phenotype were younger (60 years (49–72)), while those in the core symptoms, fatigue, and confusion (79 years (70–85)) or confusion sub-phenotypes (82 years (75–88)), were older (Fig. 2b). The GI symptoms sub-phenotype also had the highest proportion of female patients (628 (47%)). These differences were accompanied by variations in the median time from symptom onset to hospital admission between sub-phenotypes (p < 0.001) (Fig. 3a), and similarly, in the burden of co-morbidity (Fig. 3b).

To quantify these differences, we used a multinomial logistic regression model. Taking the largest sub-phenotype (core symptoms) as our reference, we included major demographic variables and co-morbidities in the analysis (Fig. 3c and Table S3). By comparison, those in the GI symptoms (RRR 0.66, 95% CI 0.60 to 0.73, p < 0.001), afebrile (RRR 0.84, 95% CI 0.76 to 0.92, *p* < 0.001), confusion (RRR 0.79, 95% CI 0.70 to 0.90, p < 0.001), and pauci-symptomatic (RRR 0.78, 95% CI 0.72 to 0.86, *p* < 0.001) sub-phenotypes, were more likely to be female (Fig. 3c). Patients assigned to the productive cough sub-phenotype were more likely to suffer from chronic pulmonary diseases (RRR 2.07, 95% CI 1.84 to 2.33, p < 0.001) and asthma (RRR 1.56, 95% CI 1.37 to 1.77, p < 0.001) (Fig. 3c).

#### Association between sub-phenotype and outcome

**Figure 4:**
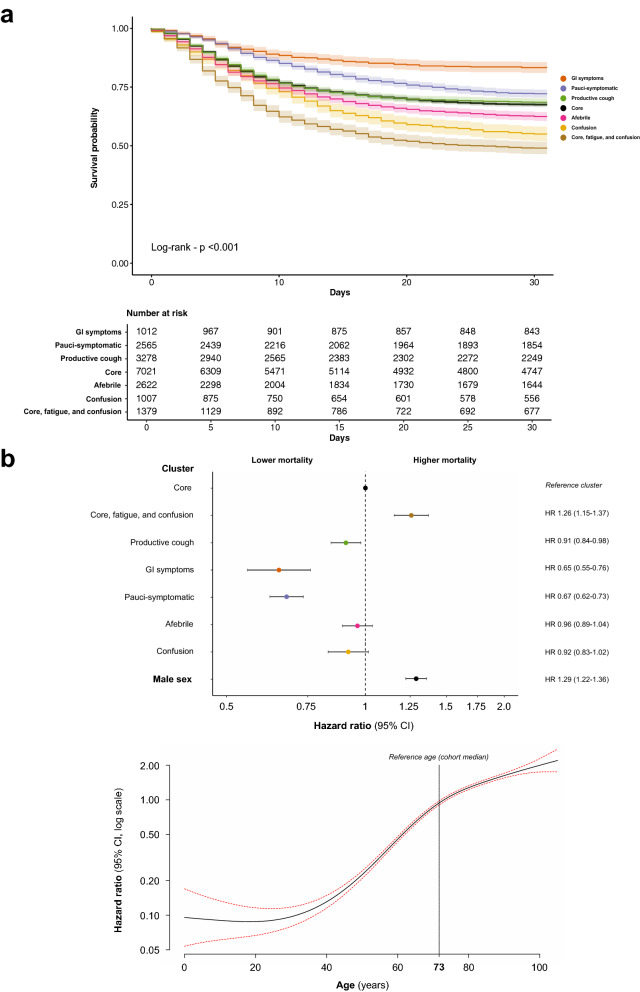
Symptom clusters and survival. (**a**) Unadjusted 30-day in-hospital mortality by cluster. Kaplan-Meier curves, those discharged before 30 days were assumed to have survived until the end of follow-up. (**b**) The risk of 30 day in-hospital mortality, adjusted for age and sex. Upper panel, Forest plot, showing results of a Cox proportional hazards model. Lower panel, Hazard ratio associated with varying age, fitted with psplines. Data are presented as hazard ratio (HR) and 95% confidence intervals. Red dotted line - 95% CI Core symptoms serves as the reference symptom cluster. The cohort median age serves as the reference age.

Overall, the unadjusted in-hospital mortality was 35%, with 24% having an incomplete hospital episode at the end of follow-up (a further 474 patients were excluded due to conflicting outcome data leaving 18,884 available for analysis). Outcomes, stratified by sub-phenotype, are detailed in Table S1. To assess differences in mortality between sub-phenotypes, we first compared Kaplan–Meier curves (log-rank test, p < 0.001) (Fig. 4a). For the largest sub-phenotype, core symptoms, unadjusted in-hospital mortality was 33%. The lowest mortality was found in the GI symptoms sub-phenotype (18%) and the highest in the core, fatigue, and confusion sub-phenotype (53%). Subsequently, we used a Cox proportional hazards model to account for the influence of age and sex (Fig. 3b). Those in the core, fatigue, and confusion sub-phenotype remained at the highest risk of death when compared to those with core symptoms, (HR 1.26, 95% CI 1.15–1.37, p < 0.001). However, membership of the confusion cluster was no longer associated with an increased risk of death (HR 0.92, 95% CI 0.83–1.02, p = 0.096). Those in the productive cough, GI, and pauci-symptomatic sub-phenotypes continued to attract a lower risk of death (Fig. 4b). Given that there was evidence of variation in risk over time for some sub-phenotypes, we performed Restricted Mean Survival Time (RMST) analyses (Table S4). By this method, males in the core, fatigue, and confusion sub-phenotype had the poorest survival compared to core symptoms, mean survival difference at 30-days -1.9 days (95% CI -2.8—1.1).

## Discussion

This study identifies distinct symptom sub-phenotypes in a large cohort of hospitalised patients with COVID-19. These sub-phenotypes are internally robust and reproducible. This report also provides one of the largest datasets of symptom prevalence in hospitalised patients with COVID-19 to date. Knowledge of distinct symptom sub-phenotypes has potential importance for our understanding of COVID-19. Two groups in our analysis have distinct clinical trajectories: those presenting with GI symptoms, and those presenting with confusion. Those in the GI cluster tended to be younger, more likely female, presented to hospital later, and had a higher probability of survival. Conversely, those with confusion (with or without fever, cough, and dyspnoea), were older, presented earlier, and had poorer outcomes. These data may be important for refining risk-prediction at the time of hospital admission. Similarly, the identification of a sub-phenotype in which patients had few symptoms other than confusion has implications for defining cases and for targeting testing, particularly in elderly patients. Importantly, these clusters have divergent outcomes from those with core COVID-19 symptoms.

The pooled symptom prevalences reported in large meta-analyses of independent studies^11,12^ are broadly consistent with ours, considering the higher severity of illness and more advanced age in our cohort. However, the prevalence of GI symptoms and of confusion was higher in our study. In an enlarged international cohort study, which includes the patients used in this analysis, estimates of symptom prevalence were largely similar^13^.

Several of our sub-phenotypes are consistent with patterns highlighted in isolation by observational studies. Confusion, as the predominant symptom, has been reported in older adults presenting to hospital with COVID-19^14–16^. Similar to our findings, this pattern has been associated with a higher risk of in-hospital death. Likewise, the GI sub-phenotype identified by our study is reflective of an enteric form of COVID-19 described in various cohorts^17^. Gastrointestinal infection occurred in SARS^18^, MERS^19^, and was reported in early descriptions of COVID-19^20^. Single-cell transcriptomic analysis from ileum and colon has demonstrated the ability of SARS-CoV-2 to infect enterocytes^21^. Consistent with our findings, observational studies of patients with predominant GI symptoms have associated this presentation with a milder course of illness and better survival^22^.

These data may offer a method for predictive enrichment in clinical trials^23^. Predictive enrichment based on sub-phenotypes is expected to perform best where causal relationships exist between fundamental biological or genomic features of disease and the clinical manifestations of severe illness^24^. Similar relationships have been described in conditions as diverse as schizophrenia^25^ and asthma^26^. Sub-phenotypes based on symptoms, may when combined with other clinical characteristics, offer a means of predictive enrichment in the absence of biochemically distinguishable sub-groups or in resource poor settings or where a highly pragmatic basis for study enrolment is employed.

This study has some limitations. First, symptoms were sought only at hospital admission, potentially disposing patients to recall bias given the time between symptom onset and presentation. Similarly, patients may have elected to describe symptoms at that time or the gamut of symptoms since the onset of illness. Additionally, a small number of symptoms now known to be associated with COVID-19, namely anosmia and ageusia^27^, were not recorded. Therefore, these data may not be generalisable to individuals with milder disease who do not require admission to hospital. Second, the patients analysed in this study were admitted in the ‘first wave’ of COVID-19, prior to the emergence of alternative variants of SARS-CoV-2, thus limiting its generalisability. However, large-scale analysis failed to find a difference in reported symptoms after the emergence of the B.1.1.7 variant in the UK^28^. Third, our study had missing data. In handling missing symptom records, we sought to retain as much data as possible given its informative missingness. This approach was robust to a sensitivity analysis. For survival data this was more challenging, and several methods of analysis were employed to reduce the risk of bias. Fourth, a limitation of partitional clustering is the need to pre-specify the number of clusters. In a large, heterogeneous population, this requires investigators to strike a balance between parsimony and granularity. Each patient is unique, and there were 4,335 symptom combinations in our cohort; hence, the most granular clustering would reveal 4,335 distinct patterns of disease. Our purpose in grouping these patients is to reveal clinical patterns that will have practical utility. As we have argued previously, the question should not be "How many clusters exist?", but rather, "Which clusters are potentially useful?"^24^. The results of our clustering analysis may also have been confounded by structural issues in the data, heterogeneity of symptom recording and differences in time from symptom onset to study enrolment being two examples. Where these were recognisable, we performed sensitivity analyses. Finally, statistical modelling in this study corrected for a limited number of co-variates. With respect to differences in patient characteristics and symptom cluster, it may be that unmodelled demographic or clinical variables account partially or wholly for the variations which we observed. Likewise, for survival analysis, we adjusted only for age and sex, both of which we know to have been largely complete and not subject to significant confounding. Additionally, for some clusters there was evidence of variation in risk of death with time. This violates the assumption of proportional hazards and may limit the interpretation of the log-rank test and hazard ratios. We attempted to defend against this violation by performing RMST analyses, which confirmed the directions of effect.

In summary, our study of 59,001 hospitalised patients with COVID-19 identified distinct symptom sub-phenotypes whose character and outcome differed significantly from those with the core symptoms of COVID-19. These observations improve our understanding of COVID-19 and have implications for clinical diagnosis, risk prediction, and future mechanistic and clinical studies.

## Methods

### Study design, setting, and population

The ISARIC Coronavirus Clinical Characterisation Consortium (4C) study is an ongoing prospective cohort study, involving 260 acute hospital sites in England, Scotland, and Wales. The study builds on an international consensus protocol for investigation of new infectious diseases, the International Severe Acute Respiratory Infection Consortium/World Health Organisation Clinical Characterisation Protocol (ISARIC/WHO CCP)^29^, designed to enable internationally harmonised clinical research during outbreaks^30^. The protocol, revision history, case report form, information leaflets, consent forms, and details of the Independent Data and Material Access Committee are available at https://isaric4c.net. The UK study was approved by the South Central—Oxford C Research Ethics Committee (13/SC/0149) and by the Scotland A Research Ethics Committee (20/SS/0028). The study was conducted in accordance with the Declaration of Helsinki 1964 and all its subsequent amendments. Informed consent to participate was obtained from all patients or an appropriate consultee. This study is reported in compliance with the TRIPOD guidelines^31^.

Patients included in the primary cohort were admitted to hospital between 6^th^ February and 8^th^ May, 2020. Inclusion criteria were all patients admitted to a participating hospital with laboratory proven or clinically highly suspected SARS-CoV-2 infection. Reverse transcription-PCR was the sole method of testing available during the study period. In the original CCP, the inclusion of patients with clinically suspected infection reflects the design of this study as a preparedness protocol where laboratory tests may not be available, but in the context of this outbreak in the UK, site training emphasised the importance of enrolling only laboratory-confirmed cases. Patients who were admitted to hospital for an unrelated condition but who subsequently tested positive for SARS-CoV-2 were also included.

### Data collection

Data were collected on a case report form, developed by ISARIC and WHO in advance of this outbreak. From admission, data were uploaded to an electronic database (REDCap, Vanderbilt University, US; hosted by University of Oxford, UK). We recorded demographic details as well as patient co-morbidities, in-hospital clinical course, treatments, and outcomes. The presence or absence of a pre-defined list of 25 symptoms was assessed at study enrolment. This was performed by research nurses and assistants, either by direct questioning or where this was not possible by review of electronic health records. Symptoms were: fever, cough, productive cough, haemoptysis, dyspnoea, wheeze, chest wall in-drawing, chest pain, fatigue, myalgia, joint pain, vomiting, diarrhoea, abdominal pain, headache, confusion, seizures, lymphadenopathy, ear pain, sore throat, runny nose, conjunctivitis, bleeding, rash, and skin ulceration. Similarly, the presence of pre-defined co-morbidities was recorded. These were: asthma, diabetes (type 1 and type 2), chronic cardiac disease, chronic haematological disease, chronic kidney disease, chronic neurological disease, chronic pulmonary disease (excluding asthma), dementia, HIV, malignancy, malnutrition, mild liver disease, moderate or severe liver disease, obesity, chronic rheumatological disease, and smoking history. Outcome data were collected for admission to critical care (Intensive Care Unit or High Dependency Unit), the use of invasive mechanical ventilation (IMV), and in-hospital mortality.

### Statistical analysis

Continuous data are summarised as median (inter-quartile range). Categorical data are summarized as frequency (percentage). Prevalence is reported as percentage (95% confidence interval). Confidence intervals were calculated for a binomial proportion using the Clopper-Pearson exact method. We analysed data using R (R Core Team, Version 4.0.0, Vienna, Austria). P values < 0.05 were deemed significant.

*Missing data—*Given the extraordinary circumstances in which this study was conducted there was a large amount of missing data. No attempt at multiple imputation was made. In respect of symptom data, in many cases the presence of a positive symptom(s) was recorded, with the remainder missing. Exploration of the structure of missing symptom data (Supplementary Figure S1) suggests that this did not occur at random. In such cases, missing symptoms were recoded as being absent i.e., recoded as ‘0’. Patients with fully missing data were excluded from the analysis.

*Symptom network analysis—*To explore the relationship between the 25 recorded symptoms we fit an Ising model, using L1-regularised logistic regression with model selection by Extended Bayesian Information criteria (EBIC)^32^, using the R package *IsingFit* (version 0.3.1). A λ value of 10 was chosen to minimise spurious conditional dependencies. The λ value acts as a tuning parameter, when multiplied by the sum of squares of the coefficients in the model, to produce a ‘shrinkage’ penalty. The partition of symptoms into communities was formalised using a short random walks method, with the R package *igraph* (version 1.2.6)^33^.

*Unsupervised partitional clustering—*Symptom data for each patient, encoded as binary responses, were used to derive a Jaccard distance matrix. This was then supplied as the input to a k-medoids clustering algorithm. This is an unsupervised partitional algorithm that seeks to divide the sample into *k* clusters, where the arbitrary distance between any individual case and the case chosen as the centre of a cluster (medoid) is minimised. In this study we used a variation of this algorithm, Clustering for Large Applications (CLARA), with the R package *cluster* (version 2.1.0)^34^. CLARA, for the optimisation of computational runtime, performs iterations of k-medoids clustering on subsets of the data and selects the best performing result. We clustered 100 random sub-samples each consisting of approximately 10% of the analysed population (n = 2,500). Each sub-sample was used to select *k* medoid cases, after which every case in the dataset was assigned to the nearest medoid. The iteration in which the mean of the dissimilarities between cases and the nearest medoid was lowest was selected. Random sampling was performed deterministically to ensure consistency between our primary analysis and validation steps. Clustering was performed agnostic of patient demographics or outcome.

The optimal number of clusters to specify to the algorithm was derived from a 'majority' assessment of three measures; total within sum of squares, average silhouette width, and gap statistic^35^, in which a parsimonious solution was preferred. To assess the stability of clusters, we employed a non-parametric bootstrap-based strategy using the R package *fpc* (version 2.2.8)^36^. This generated 1000 new datasets by randomly drawing samples from the initial dataset with replacement and applying the same clustering technique to each. Clustering results were then compared for each cluster identified in the primary analysis and the most similar cluster identified for each random re-sampling. A mean value for the Jaccard coefficient, for the sum of the comparisons, was generated for each cluster present in the primary analysis. As a sensitivity analysis for our treatment of missing data, clustering was repeated on patients with only fully complete symptom data. Cluster allocations for individuals partitioned by both iterations were then compared using Cohen's kappa and simple percentage agreement with the R package *irr* (version 0.84.1). We also performed sensitivity analyses for the effect of age, recruitment site, and time from symptom onset to study enrolment.

#### Replication

We replicated our clustering in two independent datasets, one internal and one external. Internally, we used symptom data for patients enrolled to ISARIC CCP-UK after those included in the primary cohort and until 7^th^ July, 2020 (secondary cohort). These data were processed and analysed as for the primary cohort. Missing data were treated in the same fashion. Externally, our clustering strategy was replicated independently in a sub-sample of users from the COVID Symptom Study app (developed by Zoe Global Ltd. with input from scientists and clinicians from King's College London and Massachusetts General Hospital). Individuals with confirmed SARS-CoV-2 laboratory results, registering healthy on the app, with symptom duration of more than 7 days were included, considering the presentation at symptom peak to build the clusteringy^37^.

#### Multinomial regression modelling

To quantify the relationship between demographic factors, co-morbidities, and cluster membership we built a multinomial logistic regression model with the R package *nnet* (version 7.3.14). For binary variables, a missing value was assumed to correspond to the absence of a given co-morbidity. Individuals with missing values for age and sex were excluded from this analysis. The dependent variable was symptom cluster. The independent variables were; sex, age (categorical), chronic cardiac disease, chronic pulmonary disease, asthma, chronic kidney disease, chronic neurological disease, malignancy, chronic haematological disease, HIV, chronic rheumatological disease, dementia, malnutrition, chronic liver disease, and diabetes. Results are summarised as relative risk ratio (RRR) and 95% CI. The average time from self-reported symptom onset to hospital admission in each cluster were compared using the Kruskal–Wallis test.

#### Survival analysis

To examine survival differences between symptom clusters we employed several methods. Kaplan–Meier curves for 30-day in-hospital mortality were calculated and compared using a log-rank test, with the R package *survival* (version 3.2.7). Individuals reported as being dead but with no outcome date were excluded. All individuals not reported as dead were presumed alive. Discharged individuals were retained within the at-risk set until the end of follow-up; thus, discharge was not a competing risk for death. Survival time was defined as the time in days between hospital admission and the reported outcome date. We then fitted a Cox proportional hazards model to the data with the a priori inclusion of age and sex as co-variates, using the R package *survival*. Given the non-linear effect of age on the risk of death we fitted age with a penalised smoothing spline. Results are reported as hazard ratio (HR) and 95% CI. In anticipation that the risk of death per cluster varied over time, we also calculated restricted mean survival times at 30 days, insuring the analysis against violations of proportional hazards assumptions. This was performed with the R package *survRM2* (version 1.0.3). Results are reported as mean survival difference (days) and 95% CI.

## Supplementary Information

Supplementary Information.
