# Supplementary material for: Distinct clinical symptom patterns in patients hospitalised with COVID-19 in an analysis of 59,011 patients in the ISARIC-4C study
Source: Sci Rep. 2022 Apr 27;12:6843. doi: 10.1038/s41598-022-08032-3 (PMC9043502; doi:10.1038/s41598-022-08032-3)
Supplement: Supplementary file 1 — Supplementary Information. [file 41598_2022_8032_MOESM1_ESM.docx]

**Distinct clinical symptom patterns in patients hospitalised with COVID-19 in an analysis of 59,001 patients in the ISARIC-4C study.**

Jonathan E Millar, Lucile Neyton, Sohan Seth, Jake Dunning, Laura Merson, Srinivas Murthy, Clark D Russell, Sean Keating, Maaike Swets, Carole H Sudre, Timothy D Spector, Sebastien Ourselin, Claire J Steves, Jonathan Wolf, Annemarie B Docherty, Ewen M Harrison, Peter JM Openshaw, Malcolm G Semple, J. Kenneth Baillie, on behalf of ISARIC-4C.

**Supplement**

Acknowledgements

ISARIC4C Investigators

Figure S1. Missing symptom data for the primary cohort

Figure S2. Symptom proportions and patterns by age for the primary cohort

Figure S3. Measures of optimal cluster number for the primary cohort

Figure S4. Primary cohort clustering post-hoc diagnostics and complete cases sensitivity analysis

Figure S5. Primary cohort symptom proportion by site

Figure S6. Primary cohort cluster proportion by symptom onset to study enrolment decile

Figure S7. Clustering replication in the secondary cohort

Figure S8. Clustering replication in the COVID Symptom Study

Table S1. Patient characteristics and outcomes for the primary cohort

Table S2. Symptom prevalence for the primary cohort

Table S3. Primary cohort age sensitivity analysis (individuals aged ≥ 70 years, n = 13,586)

Table S4. Multinomial regression model results

Table S5. Restricted mean survival times adjusted for age

**Acknowledgements**

The study protocol is available at <http://isaric4c.net/protocols>.

This work uses data provided by patients and collected by the NHS as part of their care and support #DataSavesLives. We are extremely grateful to the frontline NHS clinical and research staff and volunteer medical students who collected these data in challenging circumstances; and the generosity of the patients and their families for their individual contributions in these difficult times. We also acknowledge the support of Jeremy J Farrar, Nahoko Shindo, Devika Dixit, Nipunie Rajapakse, Piero Olliaro, Lyndsey Castle, Martha Buckley, Debbie Malden, Katherine Newell, Kwame O’Neill, Emmanuelle Denis, Claire Petersen, Scott Mullaney, Sue MacFarlane, Chris Jones, Nicole Maziere, Katie Bullock, Emily Cass, William Reynolds, Milton Ashworth, Ben Catterall, Louise Cooper, Terry Foster, Paul Matthew Ridley, Anthony Evans, Catherine Hartley, Chris Dunn, D Sales, Diane Latawiec, Erwan Trochu, Eve Wilcock, Innocent Gerald Asiimwe, Isabel Garcia-Dorival, J Eunice Zhang, Jack Pilgrim, Jane A Armstrong, Jordan J Clark, Jordan Thomas, Katharine King, Katie Neville, Alexandra Ahmed, Krishanthi S Subramaniam, Lauren Lett, Laurence McEvoy, Libby van Tonder, Lucia Alicia Livoti, Nahida S Miah, Rebecca K Shears, Rebecca Louise Jensen, Rebekah Penrice-Randal, Robyn Kiy, Samantha Leanne Barlow, Shadia Khandaker, Soeren Metelmann, Tessa Prince, Trevor R Jones, Benjamin Brennan, Agnieska Szemiel, Siddharth Bakshi, Daniella Lefteri, Maria Mancini, Julien Martinez, Angela Elliott, Joyce Mitchell, John McLauchlan, Aislynn Taggart, Oslem Dincarslan, Annette Lake, Claire Petersen, Scott Mullaney, and Graham Cooke.

**ISARIC4C Investigators**

Consortium Lead Investigator J Kenneth Baillie, Chief Investigator Malcolm G Semple, Co-Lead Investigator Peter JM Openshaw. ISARIC Clinical Coordinator Gail Carson. Co-Investigators: Beatrice Alex, Benjamin Bach, Wendy S Barclay, Debby Bogaert, Meera Chand, Graham S Cooke, Annemarie B Docherty, Jake Dunning, Ana da Silva Filipe, Tom Fletcher, Christopher A Green, Ewen M Harrison, Julian A Hiscox, Antonia Ying Wai Ho, Peter W Horby, Samreen Ijaz, Saye Khoo, Paul Klenerman, Andrew Law, Wei Shen Lim, Alexander, J Mentzer, Laura Merson, Alison M Meynert, Mahdad Noursadeghi, Shona C Moore, Massimo Palmarini, William A Paxton, Georgios Pollakis, Nicholas Price, Andrew Rambaut, David L Robertson, Clark D Russell, Vanessa Sancho-Shimizu, Janet T Scott, Louise Sigfrid, Tom Solomon, Shiranee Sriskandan, David Stuart, Charlotte Summers, Richard S Tedder, Emma C Thomson, Ryan S Thwaites, Lance CW Turtle, Maria Zambon. Project Managers Hayley Hardwick, Chloe Donohue, Jane Ewins, Wilna Oosthuyzen, Fiona Griffiths. Data Analysts: Lisa Norman, Riinu Pius, Tom M Drake, Cameron J Fairfield, Stephen Knight, Kenneth A Mclean, Derek Murphy, Catherine A Shaw. Data and Information System Manager: Jo Dalton, Michelle Girvan, Egle Saviciute, Stephanie Roberts Janet Harrison, Laura Marsh, Marie Connor. Data integration and presentation: Gary Leeming, Andrew Law, Ross Hendry. Material Management: William Greenhalf, Victoria Shaw, Sarah McDonald. Local Principal Investigators: Kayode Adeniji, Daniel Agranoff, Ken Agwuh, Dhiraj Ail, Ana Alegria, Brian Angus, Abdul Ashish, Dougal Atkinson, Shahedal Bari, Gavin Barlow, Stella Barnass, Nicholas Barrett, Christopher Bassford, David Baxter, Michael Beadsworth, Jolanta Bernatoniene, John Berridge , Nicola Best , Pieter Bothma, David Brealey, Robin Brittain-Long, Naomi Bulteel, Tom Burden , Andrew Burtenshaw, Vikki Caruth, David Chadwick, Duncan Chambler, Nigel Chee, Jenny Child, Srikanth Chukkambotla, Tom Clark, Paul Collini , Graham Cooke, Catherine Cosgrove, Jason Cupitt, Maria-Teresa Cutino-Moguel, Paul Dark, Chris Dawson, Samir Dervisevic, Phil Donnison, Sam Douthwaite, Ingrid DuRand, Ahilanadan Dushianthan, Tristan Dyer, Cariad Evans , Chi Eziefula, Chrisopher Fegan, Adam Finn, Duncan Fullerton, Sanjeev Garg, Sanjeev Garg, Atul Garg, Jo Godden, Arthur Goldsmith, Clive Graham, Elaine Hardy, Stuart Hartshorn, Daniel Harvey, Peter Havalda, Daniel B Hawcutt, Maria Hobrok, Luke Hodgson, Anita Holme, Anil Hormis, Michael Jacobs, Susan Jain, Paul Jennings, Agilan Kaliappan, Vidya Kasipandian, Stephen Kegg, Michael Kelsey, Jason Kendall, Caroline Kerrison, Ian Kerslake, Oliver Koch, Gouri Koduri, George Koshy , Shondipon Laha, Susan Larkin, Tamas Leiner, Patrick Lillie, James Limb, Vanessa Linnett, Jeff Little, Michael MacMahon, Emily MacNaughton, Ravish Mankregod, Huw Masson , Elijah Matovu, Katherine McCullough, Ruth McEwen , Manjula Meda, Gary Mills , Jane Minton, Mariyam Mirfenderesky, Kavya Mohandas, Quen Mok, James Moon, Elinoor Moore, Patrick Morgan, Craig Morris, Katherine Mortimore, Samuel Moses, Mbiye Mpenge, Rohinton Mulla, Michael Murphy, Megan Nagel, Thapas Nagarajan, Mark Nelson, Igor Otahal, Mark Pais, Selva Panchatsharam, Hassan Paraiso, Brij Patel, Justin Pepperell, Mark Peters, Mandeep Phull , Stefania Pintus, Jagtur Singh Pooni, Frank Post, David Price, Rachel Prout, Nikolas Rae, Henrik Reschreiter, Tim Reynolds, Neil Richardson, Mark Roberts, Devender Roberts, Alistair Rose, Guy Rousseau, Brendan Ryan, Taranprit Saluja, Aarti Shah, Prad Shanmuga, Anil Sharma, Anna Shawcross, Jeremy Sizer, Richard Smith, Catherine Snelson, Nick Spittle, Nikki Staines , Tom Stambach, Richard Stewart, Pradeep Subudhi, Tamas Szakmany, Kate Tatham, Jo Thomas, Chris Thompson, Robert Thompson, Ascanio Tridente, Darell Tupper - Carey, Mary Twagira, Andrew Ustianowski, Nick Vallotton, Lisa Vincent-Smith, Shico Visuvanathan , Alan Vuylsteke, Sam Waddy, Rachel Wake, Andrew Walden, Ingeborg Welters, Tony Whitehouse, Paul Whittaker, Ashley Whittington, Meme Wijesinghe, Martin Williams, Lawrence Wilson, Sarah Wilson, Stephen Winchester, Martin Wiselka, Adam Wolverson, Daniel G Wooton, Andrew Workman, Bryan Yates, Peter Young.

**Figure S1.** Missing symptom data for the primary cohort.

a. Summary of missing symptom data by case. b. Patterns of missing symptom data. Upset plot, intersections represent the ‘top 10’ missing symptom combinations. Upper graph summarises the number of patients with each pattern. Lower graph shows the total number of patients with missing data for each symptom.

**Figure S2.** Symptom proportions and patterns by age for the primary cohort.

Symptom proportions and patterns by age. Data are presented as percentage (%) of patients exhibiting each symptom within each decile.

**Figure S3.** Measures of optimal cluster number for the primary cohort.

a. Total within sum of squares. b. Average silhouette width. c. Gap statistic. Data are presented across a range of solutions from k = 1 to k = 10. Vertical dashed line represents the chosen solution.

**Figure S4.** Primary cohort clustering post-hoc diagnostics and complete cases sensitivity analysis.

a. Measures of clustering internal validity and stability for k = 7. * - average dissimilarity between observations in the cluster and the cluster medoid. † - isolation maximal dissimilarity between observations in the cluster and the cluster medoid/minimal dissimilarity between the cluster medoid and the medoid of any other cluster. A smaller ratio suggests the cluster is well isolated. ‡ - Jaccard bootstrap, the clusterwise Jaccard coefficient for 1000 iterations of the clustering algorithm on random subsets of the data. c. Sensitivity analysis for missing data. Clusters and patterns of symptoms for patients with fully complete symptom data (n = 12,712). Data are presented as count and percentage (%).


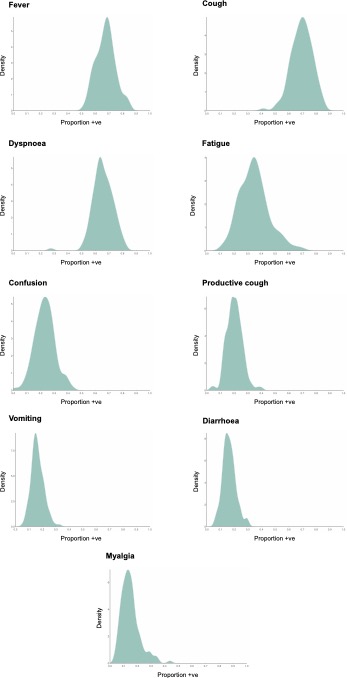


**Figure S5.** Primary cohort symptom proportion by site.

Density plots of the proportion of patients positively reporting a symptom by site (sites enrolling ≥ 50 patients, 122 sites, n = 24,279). The choice of symptoms represents the ‘top 9” symptoms reported by ≥ 50% of patients in any given cluster.

**Figure S6.** Primary cohort cluster proportion by symptom onset to study enrolment decile.

Symptom onset to study enrolment – median 8 days (4 – 13), range 1 – 30.

**Figure S7.** Clustering replication in the secondary cohort.

Cluster identities, proportions, and patterns for patients enrolled to ISARIC-CCP after the primary cohort and until 7^th^ July, 2020 (n = 33,534). Data are presented as count and percentage (%).

**Figure S8.** Clustering replication in the COVID Symptom Study.

Symptomatic individuals with laboratory confirmed SARS-CoV-2 infection, and who logged symptoms more than twice within the first week of onset. Data are presented as count and percentage (%).

**Table S1**. Patient characteristics and outcomes for the primary cohort.

|  | **n available** | **Core** | **Core, fatigue, and confusion** | **Productive cough** | **GI symptoms** | **Pauci-symptomatic** | **Afebrile** | **Confusion** | **Overall** |
| --- | --- | --- | --- | --- | --- | --- | --- | --- | --- |
| **n** (%) | | 9,364 | 1,796 | 4,234 | 1,327 | 3,692 | 3,624 | 1,440 | 25,477 |
| **Age** (years) | 23,493 | 68 (55 – 82) | 69 (55 – 81) | 72 (58 – 81) | 60 (49 – 72) | 76 (63 – 85) | 76 (64 – 85) | 82 (75 – 88) | 73 (59 – 83) |
| **Sex** (male) | 25,359 | 5,735 (61) | 1,106 (62) | 2,600 (62) | 699 (53) | 2,023 (55) | 2,084 (58) | 789 (55) | 15,046 (59) |
| **Ethnicity** (n (%)) | 22,643 |  | | | | | | | |
| Aboriginal/First Nations | | 2 (< 0.1) | 0 | 1 (< 0.1) | 0 | 1 (< 0.1) | 2 (< 0.1) | 1 (< 0.1) | 7 (< 0.1) |
| Arab | | 55 (1) | 6 (< 1) | 19 (1) | 4 (< 1) | 14 (< 1) | 16 (1) | 3 (< 1) | 117 (10 |
| Black | | 351 (4) | 46 (3) | 163 (4) | 40 (3) | 119 (4) | 87 (3) | 31 (2) | 837 (4) |
| East Asian | | 100 (1) | 16 (1) | 24 (1) | 13 (1) | 29 (1) | 23 (1) | 6 (1) | 211 (1) |
| South Asian | | 412 (5) | 50 (3) | 167 (4) | 71 (6) | 102 (3) | 116 (4) | 18 (1) | 936 (4) |
| West Asian | | 36 (< 1) | 3 (< 1) | 14 (< 1) | 5 (< 1) | 11 (< 1) | 12 (< 1) | 7 (1) | 88 (< 1) |
| Latin American | | 17 (< 1) | 1 (< 0.1) | 9 (< 1) | 2 (< 1) | 7 (< 1) | 5 (< 1) | 1 (< 0.1) | 42 (< 1) |
| White | | 6,592 (80) | 1,441 (81) | 3,100 (82) | 960 (81) | 2,807 (85) | 2,578 (86) | 1,166 (91) | 18,824 (83) |
| Other | | 687 (8) | 94 (6) | 266 (7) | 91 (8) | 194 (6) | 194 (6) | 55 (4) | 1,581 (7) |
| **Co-morbidities** (n (%)) | | | | | | | | | |
| Chronic cardiac disease | 23,933 | 2,243 (25) | 648 (38) | 1,289 (32) | 244 (19) | 1,090 (32) | 1,302 (39) | 602 (44) | 7,418 (31) |
| Diabetes | 23,670 | 1,701 (20) | 395 (23) | 724 (18) | 238 (19) | 625 (18) | 723 (22) | 266 (20) | 4,672 (20) |
| Obesity | 21,707 | 913 (11) | 146 (9) | 480 (13) | 193 (16) | 200 (6) | 291 (10) | 65 (5) | 2,288 (11) |
| Malnutrition | 22,511 | 119 (1) | 69 (4) | 67 (2) | 21 (2) | 105 (3) | 79 (3) | 66 (5) | 526 (2) |
| Chronic pulmonary disease, not asthma | 23,828 | 1,229 (14) | 351 (21) | 1,118 (28) | 128 (10) | 477 (14) | 780 (24) | 223 (17) | 4,306 (18) |
| Asthma | 23,732 | 1,279 (15) | 177 (10) | 812 (20) | 205 (16) | 297 (9) | 444 (14) | 129 (10) | 3,343 (14) |
| Chronic renal disease | 23,685 | 1,238 (14) | 342 (20) | 571 (14) | 126 (10) | 653 (19) | 610 (19) | 310 (23) | 3,850 (16) |
| Moderate/severe liver disease | 23,481 | 120 (1) | 41 (2) | 57 (1) | 18 (1) | 69 (2) | 69 (2) | 41 (3) | 415 (2) |
| Mild liver disease | 23,431 | 107 (1) | 39 (2) | 73 (2) | 25 (2) | 41 (1) | 59 (2) | 16 (1) | 360 (2) |
| Chronic neurological disease | 23,530 | 862 (10) | 327 (9) | 340 (9) | 55 (4) | 504 (15) | 355 (11) | 278 (21) | 2,721 (12) |
| Dementia | 23,630 | 1,050 (12) | 447 (26) | 271 (7) | 23 (2) | 650 (20) | 466 (14) | 467 (35) | 3,374 (14) |
| Chronic rheumatological disease | 23,407 | 783 (9) | 182 (11) | 403 (10) | 123 (10) | 347 (10) | 332 (10) | 167 (13) | 2,337 (10) |
| Chronic haematological disease | 23,454 | 340 (4) | 36 (5) | 169 (4) | 35 (3) | 163 (5) | 129 (4) | 52 (4) | 964 (4) |
| Malignancy | 23,487 | 701 (8) | 196 (12) | 422 (11) | 90 (7) | 410 (12) | 343 (11) | 158 (12) | 2,320 (10) |
| HIV | 23,311 | 37 (< 1) | 7 (< 1) | 22 (1) | 10 (1) | 10 (< 1) | 15 (1) | 3 (< 1) | 104 (< 1) |
| **Smoking status** (n (%)) | 18,357 |  | | | | | | | |
| Former smoker | | 1,935 (29) | 469 (36) | 1,232 (36) | 345 (32) | 690 (28) | 859 (34) | 308 (33) | 5,838 (32) |
| Never smoked | | 4,396 (66) | 735 (57) | 1,879 (56) | 702 (64) | 1,603 (64) | 1,432 (58) | 556 (60) | 11,321 (62) |
| Smoker | | 362 (5) | 85 (7) | 235 (7) | 44 (4) | 207 (8) | 195 (8) | 70 (8) | 1,198 (6) |
| **Outcomes** (n (%)) | | | | | | | | | |
| ICU admission | 24,443 | 1,841 (21) | 224 (13) | 700 (17) | 293 (23) | 266 (8) | 453 (13) | 69 (5) | 3,846 (16) |
| Mechanical ventilation | 23,907 | 1,073 (12) | 468 (13) | 137 (8) | 153 (12) | 130 (4) | 268 (8) | 35 (3) | 2,174 (9) |
| Mortality | 19,358 | 2,402 (33) | 741 (53) | 1,104 (33) | 186 (18) | 830 (31) | 1,086 (40) | 502 (48) | 6,851 (35) |
| Unknown mortality | 6,119 | 2,191 (23) | 389 (22) | 889 (21) | 297 (22) | 1,029 (28) | 920 (25) | 404 (28) | 6,119 (24) |

Continuous data are presented as median (inter-quartile range). HIV – human immunodeficiency virus. ICU – intensive care unit.

**Table S2**. Symptom prevalence for the primary cohort.

| **Symptom** | **n** | **Prevalence** (95% CI) |
| --- | --- | --- |
| Cough | 17,334 | 68.0 (67.5 – 68.6) |
| Fever | 16,920 | 66.4 (65.8 – 67.0) |
| Dyspnoea | 16,603 | 65.2 (64.6 – 65.8) |
| Fatigue | 8,906 | 35.0 (34.4 – 35.5) |
| Confusion | 5,935 | 23.3 (22.8 – 23.8) |
| Productive cough | 5,125 | 20.1 (19.6 – 20.6) |
| Diarrhoea | 4,171 | 16.4 (15.9 – 16.8) |
| Vomiting | 4,144 | 16.3 (15.8 – 16.7) |
| Myalgia | 3,719 | 14.6 (14.2 – 15.0) |
| Chest pain | 2,925 | 11.5 (11.1 – 11.9) |
| Headache | 2,213 | 8.7 (8.3 – 9.0) |
| Abdominal pain | 2,094 | 8.2 (7.9 – 8.6) |
| Wheeze | 2,029 | 8.0 (7.6 – 8.3) |
| Sore throat | 1,731 | 6.8 (6.5 – 7.1) |
| Joint pain | 1,294 | 5.1 (4.8 – 5.4) |
| Haemoptysis | 652 | 2.6 (2.4 – 2.8) |
| Runny nose | 583 | 2.3 (2.1 – 2.5) |
| Skin ulcers | 473 | 1.9 (1.7 – 2.0) |
| Seizures | 336 | 1.3 (1.2 – 1.5) |
| Rash | 307 | 1.2 (1.1 – 1.3) |
| Bleeding | 259 | 1.0 (0.9 – 1.1) |
| Chest wall in-drawing | 254 | 1.0 (0.9 – 1.1) |
| Lymphadenopathy | 119 | 0.5 (0.4 – 0.6) |
| Ear pain | 98 | 0.4 (0.3 – 0.5) |
| Conjunctivitis | 73 | 0.3 (0.2 – 0.4) |

Confidence intervals were calculated for a binomial proportion using the Clopper-Pearson exact method. CI – confidence interval.

**Table S3**. Primary cohort age sensitivity analysis (individuals aged ≥ 70 years, n = 13,586).

|  | **Cluster** | | | | | | | |
| --- | --- | --- | --- | --- | --- | --- | --- | --- |
|  | *1* | *2* | *3* | | *4* | *5* | *6* | *7* |
| **n** (%) | 580 (4.3) | 1537 (11.3) | 4257 (31.3) | | 1521 (11.2) | 5068 (37.3) | 622 (4.6) | 1 (< 0.1) |
| **Symptom** (%) | | | |  | | | | |
| Fever | 74.1 | 58.7 | 20.1 | | 0 | 100 | 100 | 0 |
| Cough | 76.4 | 100 | 12.0 | | 100 | 77.8 | 51.6 | 0 |
| Dyspnoea | 80.0 | 50.6 | 26.4 | | 100 | 83.6 | 0 | 0 |
| Fatigue | 56.4 | 74.1 | 16.0 | | 23.2 | 27.4 | 100 | 0 |
| Confusion | 26.6 | 31.1 | 37.3 | | 24.3 | 33.1 | 44.2 | 0 |
| Productive cough | 24.8 | 88.4 | 0.4 | | 26.6 | 13.0 | 0 | 0 |
| Vomiting | 89.7 | 13.0 | 9.3 | | 6.0 | 7.5 | 16.7 | 0 |
| Diarrhoea | 91.6 | 13.7 | 7.4 | | 8.5 | 8.3 | 18.0 | 0 |
| Myalgia | 15.3 | 16.4 | 4.2 | | 6.5 | 8.4 | 17.0 | 0 |
| *Demographics* | | | | | | | | |
| **Age** (years) | 79 (74 – 84) | 81 (75 -- 86) | 83 (77 – 88) | | 82 (76 – 87) | 81 (76 – 86) | 81 (76 – 86) | 90 |
| **Sex** (male) | 262 (45.2) | 948 (61.7) | 2301 (54.1) | | 885 (58.2) | 3032 (59.8) | 343 (55.1) | 1 (100) |
| *Outcomes* | | | | | | | | |
| **ICU admission** | 51 (8.8) | 126 (8.2) | 177 (4.2) | | 110 (7.2) | 478 (9.4) | 36 (5.8) | 0 (0) |
| **Mortality** | 184 (31.7) | 591 (38.5) | 1425 (33.5) | | 650 (42.7) | 2170 (42.8) | 189 (30.4) | 0 (0) |

Data are presented as count and percentage (%) or median (inter-quartile range). Symptoms for which ≥ 50% cluster members were positive are highlighted in red. ICU – intensive care unit.

**Table S4**. Multinomial regression model results.

|  | **Cluster** | **Odds ratio** | **95% CI** | **P value** |
| --- | --- | --- | --- | --- |
| Male sex | Core | *Reference cluster* | | |
|  | Core, fatigue, and confusion | 1.02 | 0.91 – 1.14 | 0.743 |
|  | Productive cough | 1.02 | 0.94 – 1.10 | 0.660 |
|  | GI symptoms | 0.65 | 0.57 – 0.73 | < 0.001 |
|  | Pauci-symptomatic | 0.80 | 0.73 – 0.87 | < 0.001 |
|  | Afebrile | 0.85 | 0.78 – 0.92 | < 0.001 |
|  | Confusion | 0.81 | 0.72 – 0.91 | < 0.001 |
| Age 0-20 | Core | *Reference cluster* | | |
|  | Core, fatigue, and confusion | 0.28 | 0.12 – 0.64 | 0.002 |
|  | Productive cough | 0.46 | 0.30 – 0.72 | < 0.001 |
|  | GI symptoms | 0.74 | 0.42 – 1.30 | 0.295 |
|  | Pauci-symptomatic | 3.59 | 2.81 – 4.57 | < 0.001 |
|  | Afebrile | 0.97 | 0.68 – 1.37 | 0.863 |
|  | Confusion | 0.55 | 0.26 – 1.13 | 0.101 |
| Age 20-40 | Core | *Reference cluster* | | |
|  | Core, fatigue, and confusion | 0.35 | 0.24 – 0.51 | < 0.001 |
|  | Productive cough | 0.97 | 0.82 – 1.16 | 0.771 |
|  | GI symptoms | 1.34 | 1.06 – 1.70 | 0.014 |
|  | Pauci-symptomatic | 0.89 | 0.74 – 1.07 | 0.221 |
|  | Afebrile | 0.66 | 0.54 – 0.81 | < 0.001 |
|  | Confusion | 0.20 | 0.11 – 0.36 | < 0.001 |
| Age 40-60 | Core | *Reference cluster* | | |
|  | Core, fatigue, and confusion | 0.38 | 0.32 – 0.46 | < 0.001 |
|  | Productive cough | 0.83 | 0.75 – 0.93 | < 0.001 |
|  | GI symptoms | 1.30 | 1.12 – 1.51 | < 0.001 |
|  | Pauci-symptomatic | 0.48 | 0.42 – 0.55 | < 0.001 |
|  | Afebrile | 0.56 | 0.50 – 0.64 | < 0.001 |
|  | Confusion | 0.27 | 0.21 – 0.35 | < 0.001 |
| Age >80 | Core | *Reference cluster* | | |
|  | Core, fatigue, and confusion | 1.46 | 1.30 – 1.66 | < 0.001 |
|  | Productive cough | 1.08 | 0.98 – 1.19 | 0.123 |
|  | GI symptoms | 0.53 | 0.44 – 0.65 | < 0.001 |
|  | Pauci-symptomatic | 1.46 | 1.33 – 1.61 | < 0.001 |
|  | Afebrile | 1.38 | 1.26 – 1.52 | < 0.001 |
|  | Confusion | 2.11 | 1.85 – 2.41 | < 0.001 |
| Chronic cardiac disease | Core | *Reference cluster* | | |
|  | Core, fatigue, and confusion | 1.13 | 1.00 – 1.27 | 0.041 |
|  | Productive cough | 1.24 | 1.13 – 1.36 | < 0.001 |
|  | GI symptoms | 1.01 | 0.86 – 1.20 | 0.868 |
|  | Pauci-symptomatic | 1.03 | 0.93 – 1.13 | 0.569 |
|  | Afebrile | 1.35 | 1.23 – 1.48 | < 0.001 |
|  | Confusion | 1.36 | 1.20 – 1.54 | < 0.001 |
| Chronic pulmonary disease | Core | *Reference cluster* | | |
|  | Core, fatigue, and confusion | 1.25 | 1.09 – 1.43 | 0.002 |
|  | Productive cough | 2.17 | 1.97 – 2.39 | < 0.001 |
|  | GI symptoms | 0.81 | 0.66 – 1.00 | 0.054 |
|  | Pauci-symptomatic | 0.84 | 0.74 – 0.94 | 0.004 |
|  | Afebrile | 1.48 | 1.33 – 1.65 | < 0.001 |
|  | Confusion | 0.87 | 0.74 – 1.03 | 0.105 |
| Asthma | Core | *Reference cluster* | | |
|  | Core, fatigue, and confusion | 0.82 | 0.69 – 0.98 | 0.028 |
|  | Productive cough | 1.50 | 1.35 – 1.67 | < 0.001 |
|  | GI symptoms | 0.99 | 0.81 – 1.18 | 0.915 |
|  | Pauci-symptomatic | 0.62 | 0.54 – 0.72 | < 0.001 |
|  | Afebrile | 0.94 | 0.83 – 1.06 | 0.334 |
|  | Confusion | 0.78 | 0.64 – 0.95 | 0.013 |
| Chronic kidney disease | Core | *Reference cluster* | | |
|  | Core, fatigue, and confusion | 1.05 | 0.91 – 1.21 | 0.509 |
|  | Productive cough | 0.87 | 0.78 – 0.98 | 0.023 |
|  | GI symptoms | 0.90 | 0.73 – 1.11 | 0.344 |
|  | Pauci-symptomatic | 1.18 | 1.06 – 1.32 | 0.003 |
|  | Afebrile | 1.01 | 0.90 – 1.13 | 0.883 |
|  | Confusion | 1.09 | 0.94 – 1.26 | 0.276 |

|  | **Cluster** | **Odds ratio** | **95% CI** | **P value** |
| --- | --- | --- | --- | --- |
| Chronic neurological disease | Core | *Reference cluster* | | |
|  | Core, fatigue, and confusion | 1.69 | 1.45 – 1.95 | < 0.001 |
|  | Productive cough | 0.92 | 0.80 – 1.06 | 0.238 |
|  | GI symptoms | 0.51 | 0.38 – 0.68 | < 0.001 |
|  | Pauci-symptomatic | 1.37 | 1.21 – 1.56 | < 0.001 |
|  | Afebrile | 1.01 | 0.88 – 1.15 | 0.940 |
|  | Confusion | 1.76 | 1.50 – 2.06 | < 0.001 |
| Malignancy | Core | *Reference cluster* | | |
|  | Core, fatigue, and confusion | 1.21 | 1.01 – 1.44 | 0.035 |
|  | Productive cough | 1.28 | 1.11 – 1.46 | < 0.001 |
|  | GI symptoms | 1.13 | 0.89 – 1.44 | 0.324 |
|  | Pauci-symptomatic | 1.35 | 1.18 – 1.55 | < 0.001 |
|  | Afebrile | 1.13 | 0.98 – 1.30 | 0.095 |
|  | Confusion | 1.14 | 0.94 – 1.38 | 0.183 |
| Chronic haematological disease | Core | *Reference cluster* | | |
|  | Core, fatigue, and confusion | 0.99 | 0.76 – 1.30 | 0.962 |
|  | Productive cough | 1.01 | 0.83 – 1.24 | 0.885 |
|  | GI symptoms | 0.72 | 0.49 – 1.05 | 0.088 |
|  | Pauci-symptomatic | 1.00 | 0.82 – 1.23 | 0.975 |
|  | Afebrile | 0.84 | 0.68 – 1.05 | 0.125 |
|  | Confusion | 0.83 | 0.61 – 1.12 | 0.223 |
| HIV | Core | *Reference cluster* | | |
|  | Core, fatigue, and confusion | 1.03 | 0.42 – 2.57 | 0.944 |
|  | Productive cough | 1.58 | 0.91 – 2.73 | 0.103 |
|  | GI symptoms | 1.97 | 0.96 – 4.02 | 0.063 |
|  | Pauci-symptomatic | 0.95 | 0.46 – 1.95 | 0.887 |
|  | Afebrile | 1.40 | 0.75 – 2.60 | 0.291 |
|  | Confusion | 0.25 | 0.03 – 1.86 | 0.175 |
| Chronic rheumatological disease | Core | *Reference cluster* | | |
|  | Core, fatigue, and confusion | 0.99 | 0.83 – 1.19 | 0.953 |
|  | Productive cough | 1.05 | 0.91 – 1.20 | 0.509 |
|  | GI symptoms | 1.24 | 1.00 – 1.54 | 0.053 |
|  | Pauci-symptomatic | 0.97 | 0.84 – 1.11 | 0.622 |
|  | Afebrile | 0.92 | 0.80 – 1.06 | 0.256 |
|  | Confusion | 1.02 | 0.85 – 1.23 | 0.804 |
| Dementia | Core | *Reference cluster* | | |
|  | Core, fatigue, and confusion | 1.58 | 1.38 – 1.82 | < 0.001 |
|  | Productive cough | 0.48 | 0.42 – 0.56 | < 0.001 |
|  | GI symptoms | 0.22 | 0.15 – 0.34 | < 0.001 |
|  | Pauci-symptomatic | 1.15 | 1.02 – 1.29 | 0.089 |
|  | Afebrile | 0.81 | 0.72 – 0.92 | 0.001 |
|  | Confusion | 1.80 | 1.57 – 2.07 | < 0.001 |

**Table S5**. Restricted mean survival times adjusted for age.

| **Cluster** | **Sex** | **Mean difference** (days) | **95% CI** | **P value** |
| --- | --- | --- | --- | --- |
| Core | Male | *Reference cluster* | | |
|  | Female |  |  |  |
| Core, fatigue, and confusion | Male | -1.9 | -2.8 - -1.1 | < 0.001 |
|  | Female | -1.7 | -2.8 - -0.6 | 0.002 |
| Productive cough | Male | 0.5 | -0.1 – 1.0 | 0.085 |
|  | Female | 0.5 | -0.2 – 1.2 | 0.132 |
| GI symptoms | Male | 1.3 | 0.5 – 2.1 | 0.002 |
|  | Female | 2.7 | 2.0 – 3.5 | < 0.001 |
| Pauci-symptomatic | Male | 2.5 | 1.9 – 3.0 | < 0.001 |
|  | Female | 2.3 | 1.7 – 2.9 | < 0.001 |
| Afebrile | Male | 0.4 | -0.3 – 1.0 | 0.258 |
|  | Female | 0.3 | -0.4 – 1.0 | 0.426 |
| Confusion | Male | 0.7 | -0.3 – 1.7 | 0.158 |
|  | Female | 1.2 | 0.1 – 2.3 | 0.038 |

CI – confidence interval.
